# New Inventions

**Published:** 1912-12

**Authors:** 


### NEW INVENTIONS

---

J. S. Stillwell, West Orange, N. J. 1,041,896. Malted Whey.

A. H. Stone, Boston, Mass. 1,041,897. Depilatory and Process of Making it.

Kate Van Hook, Hiawatha, Kansas. 1,042,058. Massage Apparatus.

Henry Green, Hartford, Conn. 1,042,109. X-Ray Tube.

D. W. Dorrance, Koler, Oreg. 1,042,413. Artificial Hand.

Gustav Fritsche, Strzebowitz near Schonbraum,, Austria-Hungary, assignor to The Roessler & Hasslacher Chemical Co., New York, N. Y. 1,042,422. Germicide and Insecticide.

S. B. Goff, Canden, N. J. 1,042,549. Fountain Syringe.

Jas. J. Holland, Philadelphia, Pa., and Wm. Discher, Toledo, Ohio. 1,042,556. Nebulizer.

J. B. Wagoner, Los Angeles, Cal. 1,042,624. Recital Dynams.

G. J. Kelley, Attleboro, Mass. 1,042,685. Atomizer.

Chas. Goulding, Chicago, Ill. 1,042,785. Pill and Tablet Counting Machine.

J. J. Myers, E. Lansing, Mich. 1,042,815. Model of the Human Eye.

W. O. Kaiser, Burlington, Iowa. 1,042,923. Ointment Machine.

Heinrich Krane, Munder, Germany. 1,041,105. Drop Bottle.

N. E. Mighell, Marshalltown, Iowa. 1,041,138. Surgical Appliance.

Lucy Alden, Saginaw, Mich. 1,041,225. Catamenial Garment.

J. L. Bornstein, Dayton, Ohio. 1,041,420. Sanitary Support

Heinrich Thron, Frankfort-on-the-Main. 1,041,528. Mfg. of Esters of the Hydrocinchona Alkaloids.

Heinrich Hoerlein, Vohwinkel near Elberfeld, Germany, assignor to Farbenfabriken vorm Friedr. Bayer & Co., Elberfeld, Germany. 13,477. Phenylethylbaibituric acid.

Ludwig Benda, Frankfort-on-the-Main, Germany, assignor to Farbwerke vorm Meister Lucious & Bruning, Hockst-on-the-Main, Ger. 1,040,260. 2.5 Diaminobenzene-Arsenic Acid.

H. J. Collis, Taunton, Mass. 1,040,279. Ankle Support and Protector.

L. E. Knott, Boston, Mass., assignor to L. E. Knott Apparatus Co., Boston, Mass. 1.040,356. Apparatus for Determining the amount of Carbon-Dioxide in the Air.

W. E. Poindexter, Sedalis, Mo. 1,040,404. Pile Truss.

E. W. Schneider, New York, N. Y., assignor to The Mears Ear Phone Co., New York, N. Y. 1,040,428. Device for Aiding the Hearing.

E. W. Schneider, New York, N. Y. 1,040,429. Device for Aiding the Hearing.

Allen De Vilbiss, Toledo, Ohio. 1,040,523. Bone Forceps or the Like.

Francis H. Escherich, Washington, D. C. 1,040,659. Nursing Bottle Holder.

